# BMP4 and perivascular cells promote hematopoietic differentiation of human pluripotent stem cells in a differentiation stage-specific manner

**DOI:** 10.1038/s12276-019-0357-5

**Published:** 2020-01-20

**Authors:** Suji Jeong, Borim An, Jung-Hyun Kim, Hyo-Won Han, Jung-Hyun Kim, Hye-Ryeon Heo, Kwon-Soo Ha, Eun-Taek Han, Won Sun Park, Seok-Ho Hong

**Affiliations:** 1 Department of Internal Medicine, School of Medicine, Kangwon National University, Chuncheon, 24341 Republic of Korea; 2 Division of Intractable Diseases, Center for Biomedical Sciences, Korea National Institute of Health, Korea Centers for Disease Control and Prevention, Cheongju, 28159 Republic of Korea; 3 Department of Molecular and Cellular Biochemistry, School of Medicine, Kangwon National University, Chuncheon, 24341 Republic of Korea; 4 Department of Medical Environmental Biology and Tropical Medicine, School of Medicine, Kangwon National University, Chuncheon, 24341 Republic of Korea; 5 Department of Physiology, School of Medicine, Kangwon National University, Chuncheon, 24341 Republic of Korea

**Keywords:** Haematopoietic stem cells, Pluripotent stem cells

## Abstract

The efficient and reproducible derivation and maturation of multipotent hematopoietic progenitors from human pluripotent stem cells (hPSCs) requires the recapitulation of appropriate developmental stages and the microenvironment. Here, using serum-, xeno-, and feeder-free stepwise hematopoietic induction protocols, we showed that short-term and high-concentration treatment of hPSCs with bone morphogenetic protein 4 (BMP4) strongly promoted early mesoderm induction followed by increased hematopoietic commitment. This method reduced variations in hematopoietic differentiation among hPSC lines maintained under chemically defined Essential 8 medium compared to those maintained under less-defined mTeSR medium. We also found that perivascular niche cells (PVCs) significantly augmented the production of hematopoietic cells via paracrine signaling mechanisms only when they were present during the hematopoietic commitment phase. A protein array revealed 86 differentially expressed (>1.5-fold) secretion factors in PVC-conditioned medium compared with serum-free control medium, of which the transforming growth factor-β inducible gene H3 significantly increased the number of hematopoietic colony-forming colonies. Our data suggest that BMP4 and PVCs promote the hematopoietic differentiation of hPSCs in a differentiation stage-specific manner. This will increase our understanding of hematopoietic development and expedite the development of hPSC-derived blood products for therapeutic use.

## Stem cells: A better way to grow blood cells in the laboratory

Adding a vital regulatory molecule and support cells to the culture medium can help in the derivation of blood products from stem cells. A team led by Seok-Ho Hong from Kangwon National University in Chuncheon, South Korea, followed a clinical-grade protocol for converting embryonic stem cells or induced pluripotent stem cells from adults into blood cell precursors. The researchers showed that incorporating high doses of a growth factor called bone morphogenetic protein 4 into the standard culture medium for a short period promoted early differentiation toward blood cells. Incorporating so-called perivascular cells taken from umbilical cord blood also enhanced the process through the secretion of signaling molecules that further pushed the stem cells toward differentiating into blood cells. The findings could help improve protocols for making blood products from stem cells for therapeutic purposes.

## Introduction

Human embryonic stem cells (hESCs) offer an attractive alternative to the generation of adult hematopoietic stem and progenitor cells (HSPCs) due to their pluripotency and their unlimited potential to self-renew^1^. Furthermore, two pluripotent alternatives to hESCs that may be able to generate autologous HSPCs have been developed for personalized regenerative medicine: (1) ESCs derived by somatic cell nuclear transfer (SCNT) and (2) induced pluripotent stem cell (iPSC) differentiation driven by the forced expression of transcription factors^2,3^. As a consequence, various strategies have been developed over the last two decades to enhance the induction efficiency of undifferentiated human pluripotent stem cells (hPSCs) into specific lineages and improve their reproducibility^4^.

Hematopoiesis is spatiotemporally regulated by the specific milieu of hematopoietic cytokines and environmental factors that are encountered at the sites of development^5,6^. However, initial induction protocols applied a cocktail of hematopoietic cytokines continuously from early mesodermal induction to the hematopoietic commitment phase without considering the temporal regulation of these cytokines^7^. Thus, it is critical to develop a protocol that provides sequential treatment with hematopoietic cytokines relevant to hematopoietic developmental processes and the environmental factors that control HSPC maintenance and development. Recent studies have reported efficient hematopoietic differentiation from hESCs, hiPSCs and hSCNT-ESCs based on stepwise induction protocols applying sequential supplementation of different combinations of hematopoietic cytokines^8–11^. It was shown that coculture of hPSCs with mouse bone marrow (BM) stromal cells and monolayers of cells derived from the mouse aorta-gonad-mesonephros (AGM) region enhance hematopoietic differentiation^12,13^. Furthermore, several types of cells isolated from human fetal and adult tissues that provide factors involved in the development and maintenance of HSPCs have been evaluated to eliminate concern over xenogeneic contamination and thereby mimic the HSPC niche^14,15^. All these studies strongly indicate that spatiotemporal regulation should be combined to develop transplantable HSPCs from hPSCs. However, this remains a challenge due to the inability to recapitulate the developmental stages and simulate a BM microenvironment.

In this study, we used serum-, xeno- and feeder-free stepwise hematopoietic induction protocols to assess the influence of bone morphogenetic protein 4 (BMP4) and perivascular niche cells (PVCs) on the hematopoietic development of cells from hPSCs. We showed that a short induction with a high concentration of BMP4 promoted the frequency and number of cells committed to the hematopoietic lineage. Interestingly, PVCs that comprised the BM niche augmented the production of hematopoietic cells via paracrine mechanism only when they were present during the hematopoietic commitment phase but had no effect during the hemogenic specification phase. Our findings highlighted the importance of growth timing and niche factor exposure during hematopoietic development.

## Materials and methods

### Maintenance of hPSCs

Human PSCs (CAH15, hEF12-2 and iPS-NT4-S1) were kindly provided by CHA University, South Korea. Additionally, human iPSCs (CMC lines; CMC003, CMC009 and CMC011) were obtained from the Korea National Stem Cell Bank (kscr.nih.go.kr). The cells were cultured under serum- and feeder-free conditions using mTeSR1 medium (Stem Cell Technologies) on Matrigel (BD Biosciences)-coated dishes or under xeno-, serum- and feeder-free conditions using E8 medium (Stem Cell Technologies) on dishes coated with recombinant human vitronectin (Stem Cell Technologies). They were subcultured at 80–90% confluency and passaged every 4–5 days by mechanical dissociation. All cells were incubated at 37 °C in a humidified atmosphere with 5% CO_2_.

### Stepwise hematopoietic differentiation of hPSCs

Stepwise direct HSC differentiation was performed as previously described^9,11^. Undifferentiated colonies of hPSCs were prepared at a density of <5 colonies per well. When the colonies grew to ~500 µm in diameter, they were cultured in hematopoietic differentiation medium (HDM) containing Stemline II serum-free medium (Sigma), Insulin-Transferrin-Selenium (Gibco) and BMP4 (20 and 100 ng/mL) for the first 2 days. The colonies were incubated with HDM containing 20 ng/mL BMP4 for 2 days, followed by treatment with 40 ng/mL vascular endothelial growth factor and 50 ng/mL stem cell factor (SCF) for 2 days. On day 6, the cultures were given fresh HDM supplemented with hematopoietic cocktail (50 ng/mL SCF, 10 ng/mL thrombopoietin, 50 ng/mL interleukin-3 (IL-3), 50 ng/mL granulocyte colony-stimulating factor and 50 ng/mL Feline McDonough Sarcoma (FMS)-like tyrosine kinase 3 ligand (all from R&D Systems)) and cultured for 13 days. The medium was changed every 3 days. The induction efficiency of hPSCs into the hematopoietic lineage was assessed by measuring the frequencies of hemogenic precursors (CD45-CD31+), hematopoietic progenitors (CD34+CD45+ or CD34+CD43+) and mature blood cells (CD34-CD45+ and CD34-CD43+) by flow cytometry.

### Colony-forming unit assay

A colony-forming unit (CFU) assay was performed as previously described^16^. Briefly, 10,000 hematopoietic progenitor cells were seeded in methylcellulose (Stem Cell Technologies, H4434) supplemented with SCF (50 ng/mL), IL-3 (10 ng/mL), erythropoietin (3 U/mL) and granulocyte-macrophage-CSF (10 ng/mL). After incubation for 7–10 days at 37 °C in 5% CO_2_, hematopoietic cell clusters were counted on the basis of morphology.

### Flow cytometry analysis

The floating hematopoietic cells were collected after 12–18 days of differentiation. Adherent cells were incubated with collagenase B (Roche) for 2 h, followed by treatment with cell dissociation buffer (Gibco). The cells were passed through a 70-μm cell strainer and incubated with the following fluorochrome-conjugated anti-human antibodies for 1 h at 4 °C: CD31-PE (#555446), CD34-FITC (#555821), CD45-APC (#555485), and CD43-APC (#560198) (all from BD Biosciences). Dead cells were excluded based on staining with 7-aminoactinomycin D (BD Pharmingen, #559925). The expression of HSPC markers was measured using a FACSCanto^TM^II flow cytometer (BD Bioscience), and the acquired data were analyzed with FlowJo software (Tree Star).

### Real-time quantitative PCR

Total RNA from cancer cell lines was extracted with the RNeasy Mini Kit (Qiagen Inc., Valencia, CA) and converted to first-strand cDNA with a TOPscriptTM RT DryMIX kit (Enzynomix). The cDNA was amplified and quantified using AccuPower® PCR PreMix reagents (Bioneer Corp.). The primer sequences were as follows: Brachyury, forward (F): 5′-ATGAGCCTCGAATCCACATAGT-3′ and reverse (R): 5′-TCCTCGTTCTGATAAGCAGTCA-3′; MIXL1, F: 5′-GGATCCAGGTATGGTTCCAG-3′ and R: 5′-GGAGCACAGTGGTTGAGGAT-3′; GAPDH, F: 5′-TGCACCACCAACTGCTTAGC-3′ and R: 5′-GGCATGGACTGTGGTCATGAG-3′; c-KIT (receptor tyrosine kinase), F: 5′-CACCTTGGGCGAGAGCTGGAAC-3′, R: 5′-TCCTGCTGCCACACATTGGAGC-3′; Tie-2 (tyrosine protein kinase receptor), F: 5′-CCAGGATGGCAGGGGCTCCA-3′, R: 5′-GGTAGCGGCCAGCCAGAAGC-3′; and vWF (von Willebrand factor), F: 5′-GCAGTGGAGAACAGTGGTG-3′, R: 5′-GTGGCAGCGGGCAAAC-3′. The expression levels of Brachyury, MIXL1, c-KIT, Tie-2 and vWF were normalized to the level of GAPDH, and data analysis was performed by the comparative CT method.

### Isolation, characterization and differentiation of human umbilical cord-derived PVCs

Human umbilical cord (HUC)-derived PVCs were isolated and cultured as previously described^17^. HUCPVCs were obtained from full-term deliveries by cesarean section with donors’ written consent (IRB No. 2012-11-003-003; Kangwon National University Hospital). The surface markers CD146, CD44 and CD90 were tested by flow cytometry. The multilineage differentiation potential of HUCPVCs was evaluated at passages 2–4. For adipogenic differentiation, HUCPVCs were seeded at 5 × 10^3^ cells/cm^2^ in 12-well tissue culture plates and cultured in StemXVivo adipogenic medium (R&D Systems) for 21 days. On day 21, the cells were fixed with 4% PFA for 5 min and stained with Oil Red O (Lifeline Cell Technology) to visualize the intracellular accumulation of lipid vacuoles. For osteogenic differentiation, HUCPVCs were plated at 5 × 10^3^ cells/cm^2^ in 12-well tissue culture plates and cultured in StemXVivo osteogenic supplement (R&D Systems) for 21 days. On day 21, the cells were fixed in 4% PFA for 10 min and stained with Alizarin Red S (Lifeline Cell Technology) to visualize mineralization in osteogenic cell cultures. For chondrogenic differentiation, 2.5 × 10^5^ cells were centrifuged for 15 min at 1200 rpm in a 15-mL polypropylene tube. Then, the pellets were cultured with a StemPro Chondrogenesis Differentiation Kit (Gibco) for 21 days. On day 21, the pellets were fixed in 4% PFA, dehydrated, and embedded in paraffin for histology to detect sulfated proteoglycans by Alcian Blue staining.

### Preparation of conditioned medium and protein array analysis

HUCPVCs were seeded on 100-mm dishes and cultured until they reached 80–90% confluence. The cells were rinsed with PBS and then incubated with *α*-minimum essential medium without FBS for 24 h at 37 °C in a humidified atmosphere with 5% CO_2_. The conditioned medium (CM) was collected, passed through a 0.22-μm filter (Sartorius, #16534) and condensed with a 3-kDa cutoff membrane (Amicon) using an ultracentrifuge at 4 °C and 6000 × *g* for 1 h. Proteins in the condensed CM were quantified using a BCA assay kit (Thermo Scientific). The PVC-CM was stored at −70 °C and used for protein array analysis using the RayBio® L-Series Human Antibody Array 1 kit (L507 and L493, AAH-BLG-1000-2; RayBiotech, Norcross, GA) containing 1000 human proteins. Secretion factors that were expressed 1.5-fold higher or greater relative to the control were scored as significant. To determine the effects of PVC-CM on the production of hematopoietic cells from hPSCs, PVC-CM was applied on days 0–9 and 9–17 of hematopoietic differentiation.

### Statistical analysis

All data represent at least three independent experiments and are expressed as the mean ± SD unless otherwise indicated. Statistical significance was determined using Student’s *t*-test, and *p* < 0.05 was considered statistically significant.

## Results

### Short induction with a high dose of BMP4 strongly promoted undifferentiated hPSCs into the hematopoietic lineage

**Fig. 1:**
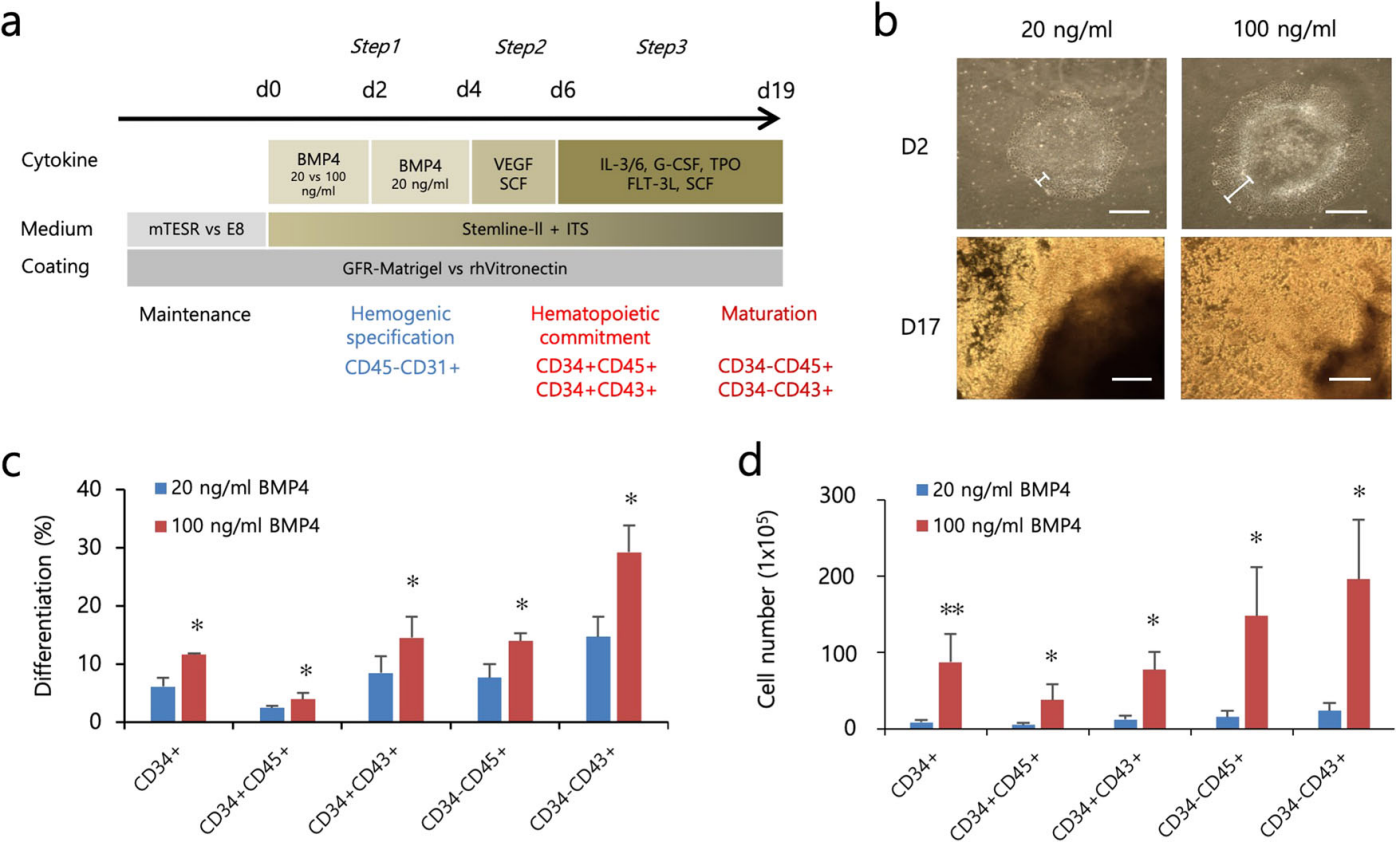
Comparison of the effects of low- and high-dose BMP4 on the hematopoietic differentiation of hPSCs. **a** A schematic overview of the stepwise hematopoietic induction protocol for hESCs (CHA15) and hiPSCs (hEF12-2 and iPS-NT4-S1). **b** Representative bright-field images of colonies at the early (day 2) and late (day 17) stages of hematopoietic differentiation with BMP4 (20 vs 100 ng/mL). Scale bar, 200 μm. **c**, **d** Effects of low- and high-dose BMP4 on the induction efficiency (**c**) and the yield (**d**) of hematopoietic lineage cells from hPSCs were analyzed by flow cytometry. The bars indicate the mean ± SD; *p < 0.05, **p < 0.01.

BMP4 plays an indispensable role in inducing undifferentiated hPSCs to the early mesodermal lineage and in forming hemangioblasts in a dose-dependent manner^18–20^. However, the temporal role of BMP4 in serum- and feeder-free hematopoietic induction conditions has not been fully evaluated. A previous report showed that short-term BMP4 treatment for 24 h sufficiently induces early mesodermal lineage, whereas long-term BMP4 treatment for up to 7 days results in trophoblast differentiation^21,22^. Therefore, we first focused our study on the mesodermal-initiating effects of short-term BMP4 treatment in hPSCs using our modified directed serum- and feeder-free stepwise hematopoietic induction protocol. We maintained one hESC line and two hiPSC lines in serum-free mTeSR medium in Matrigel-coated dishes. Upon hematopoietic induction, we administered BMP4 at low and high concentrations (20 and 100 ng/mL) initially for 2 days and investigated morphological changes and hematopoietic output on day 17 (Fig. 1a). Short-term BMP4 treatment led to morphological changes characterized by flattened and enlarged cells regardless of concentration; these changes initially appeared on the edge of colonies and subsequently extended to the center. However, colonies treated with a high concentration of BMP4 had a broader flattened area compared with that of colonies treated with a low concentration of BMP4 (Fig. 1b). We then assessed the commitment capacity of the cells treated with BMP4 by measuring the frequencies of primitive HPCs (CD34+CD45+ or CD34+CD43+) and mature blood cells (CD34−CD45+ or CD34−CD43+) on day 17. Both HPCs and mature blood cells occurred at a higher frequency in cells treated with 100 ng/mL BMP4 compared with cells treated with 20 ng/mL BMP4 (Fig. 1c). In addition, the cells treated with 100 ng/mL BMP4 yielded significantly more HPCs and mature blood cells compared with the cells treated with 20 ng/mL BMP4 (Fig. 1d). We further asked whether the length (days 0–2 vs days 0–4) of BMP4 exposure at a high concentration (100 ng/mL) influences hematopoietic induction efficiency. We found that, compared with 2 days of exposure to BMP4, a longer duration of BMP4 induction (days 0–4) did not yield any differences in the frequency or number of hematopoietic cells compared (Supplementary Fig. 1). These results demonstrated that treatment with BMP4 at a high concentration for a short time period (2 days) more robustly and sufficiently induced undifferentiated hPSCs to differentiate into hematopoietic lineages.

### Maintenance conditions affected variations in hematopoietic differentiation among hPSC lines

**Fig. 2:**
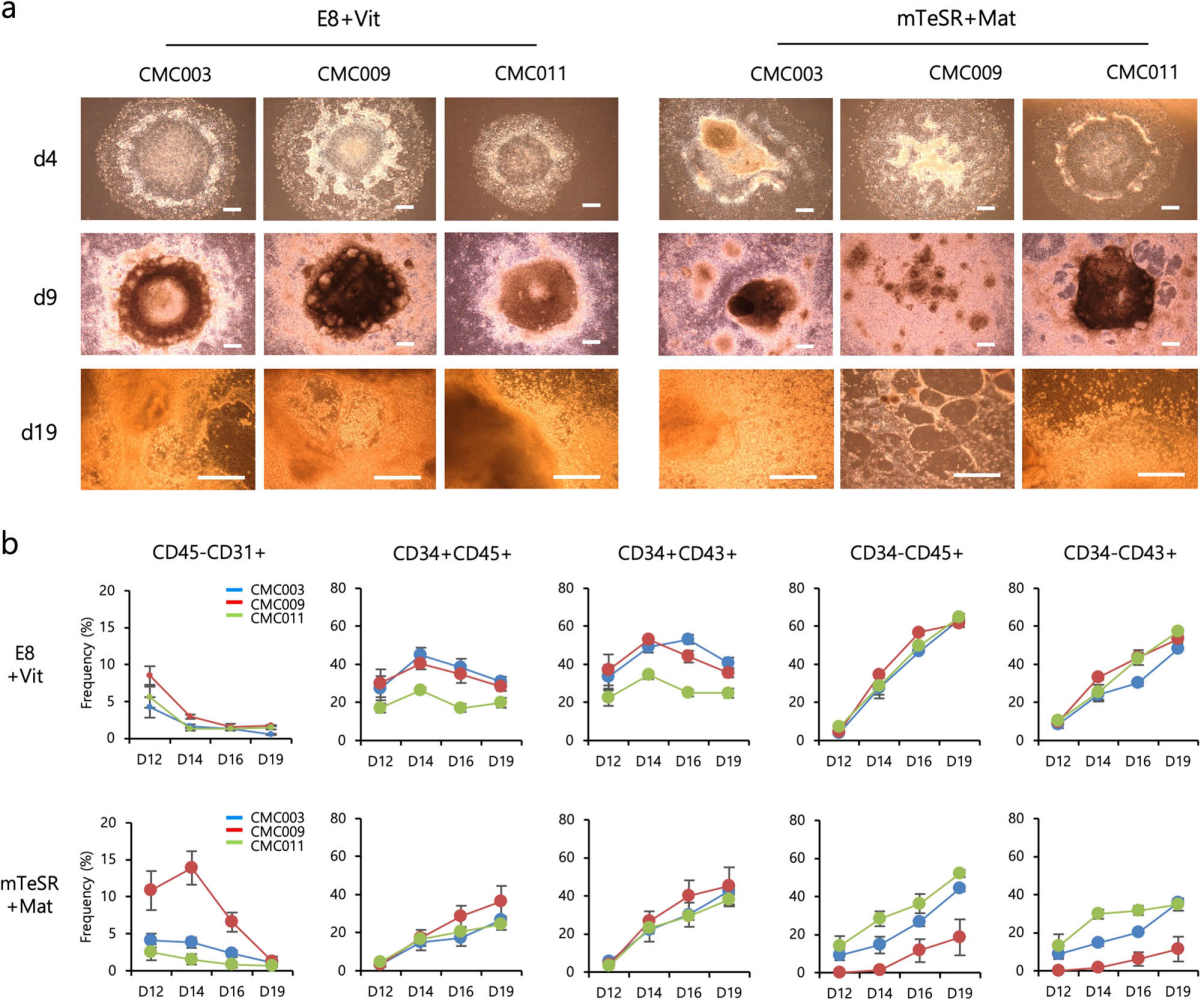
Comparison of hPSC culture conditions for the optimization of hematopoietic differentiation from hPSCs. **a** Representative bright-field images of colonies during hematopoietic differentiation of hiPSC lines (CMC003, CMC009 and CMC011) maintained in two different culture conditions (E8+Vit vs mTeSR+Mat). **b** Temporal expression patterns of hematopoietic lineage markers (CD45−CD31+, hemogenic precursors; CD34+CD45+ and CD34+CD43+, hematopoietic progenitors; CD34−CD45+ and CD34−CD43+, mature blood cells) during hematopoietic development from hiPSC lines were analyzed by flow cytometry. The bars indicate the mean ± SD.

**Fig. 3:**
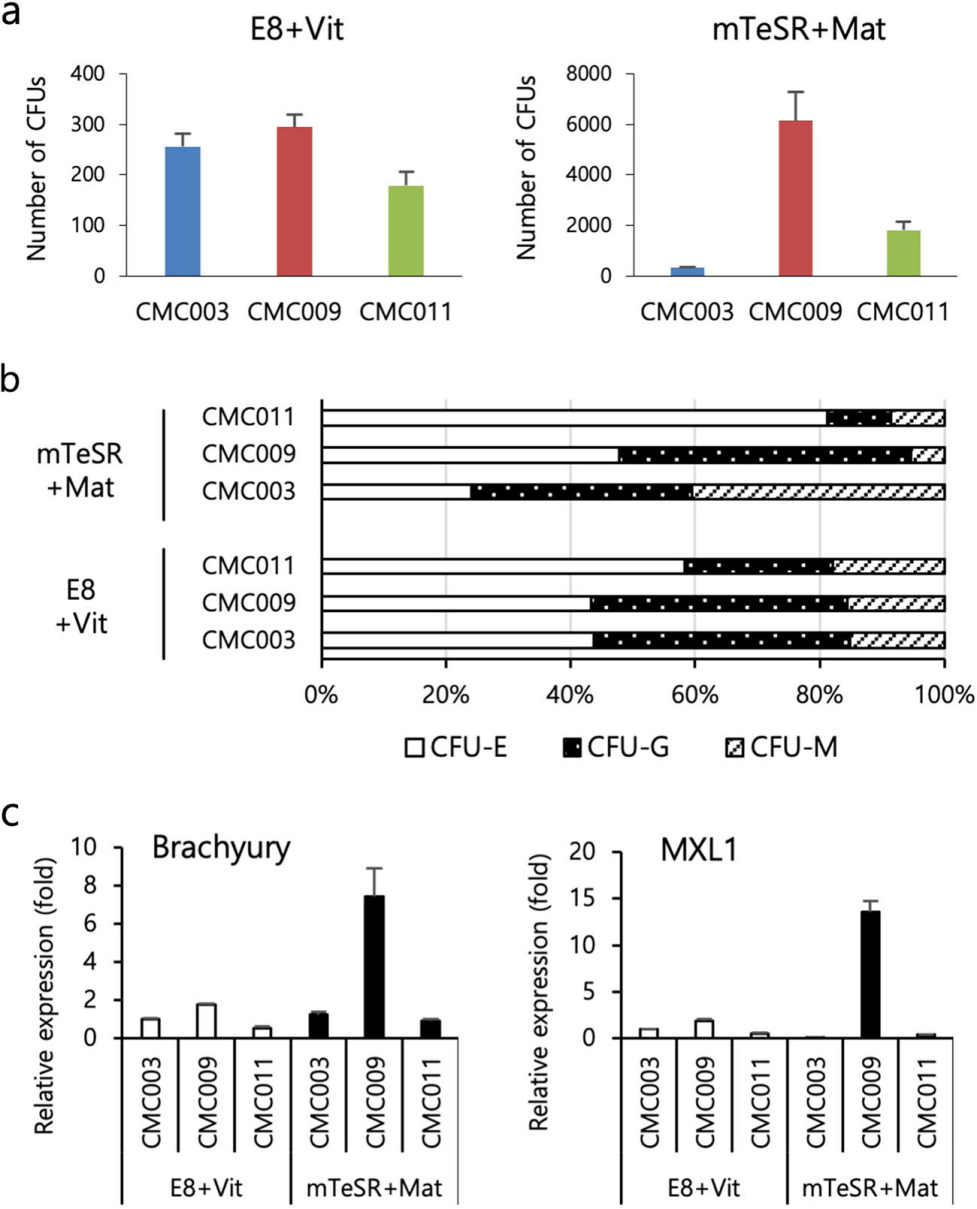
Comparison of hematopoietic progenitor capacity to generate colonies. **a** Assessment of hematopoietic progenitor capacity of hiPSCs maintained under E8 + Vit vs mTeSR + Mat culture conditions. **b** Distribution of CFU subtypes. **c** qPCR analysis for Brachyury and MIXL1 transcripts in undifferentiated hiPSC cultures. The bars indicate the mean ± SD. CFU-E, erythrocyte; CFU-G, granulocyte; CFU-M, macrophage.

To determine the reproducibility and efficacy of our protocol, we tested three independent hiPSC lines (CMC003, CMC009 and CMC011) maintained in the following two different conditions: xeno- and feeder-free E8 medium in combination with rhVitronectin XF™ (E8+Vit) and feeder-free mTeSR™ medium in combination with Matrigel® (mTeSR+Mat). During the hemogenic specification phase, the colonies exhibited a plateau-like central area and a flattened surrounding area and then formed a beaded-like structure along the edges of the plateau area. As the commitment phase progressed, floating hematopoietic cells and vessel-like structures were observed on days 12–14 and gradually increased up to day 19 (Fig. 2a). Overall, the cell lines cultured under the E8+Vit condition showed even more morphological changes than the cell lines cultured under the mTeSR+Mat condition (Fig. 2a). Next, we assessed the temporal frequency of hemogenic precursors (CD45−CD31+), HPCs and mature blood cells. The cells under both culture conditions displayed a similar rise and fall of each cell population corresponding to hematopoietic development in response to sequential stimulation by hematopoietic cytokines. In the E8+Vit condition, cell line variations in the frequency of HPCs were observed but were lower at the maturation stage. In contrast, the cell lines cultured in the mTeSR+Mat condition showed a large variation in the frequency of mature blood cells (Fig. 2b). We further assessed the hematopoietic potential of HPCs derived from both conditions using a CFU assay. Relatively low variation in the number and distribution of CFU subtypes was detected among cell lines maintained in the E8+Vit condition, whereas a wide variation was still found among cell lines maintained in the mTeSR+Mat condition (Fig. 3a, b). Notably, the expression levels of early mesodermal genes, including *Brachyury* and *MIXL1*, showed more variations in cell lines cultured in the mTeSR+Mat condition compared with those cultured in the E8+Vit condition (Fig. 3c). These results indicated that our optimized hematopoietic protocol was applicable to various cell lines and culture conditions and was more suitable for the E8+Vit culture condition in terms of reproducibility and equivalent differentiation potential in the late stage of hematopoietic development.

### PVCs augmented hematopoietic differentiation from hPSCs in a stage-specific manner via paracrine signaling mechanisms

**Fig. 4:**
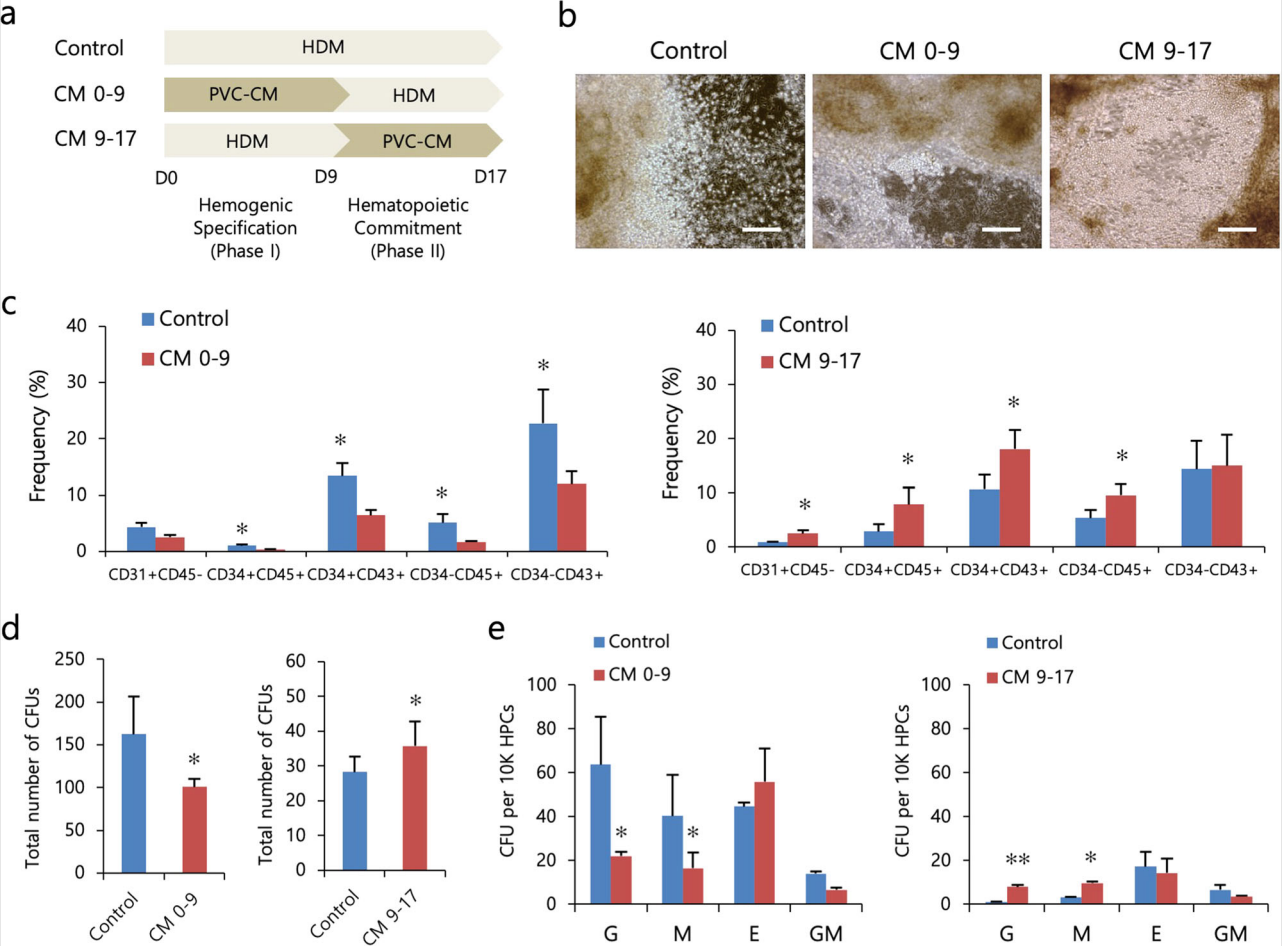
PVCs enhance the hematopoietic differentiation of hPSCs via paracrine action. **a** Experimental scheme used to determine the paracrine effects of PVCs on the hematopoietic differentiation of hPSCs (CHA15 and iPS-NT4-S1). **b** Representative bright-field images of colonies on day 17 of hematopoietic differentiation. Scale bar, 50 μm. **c** Effects of PVC-CM on the production of hematopoietic lineage cells for two different exposure times (days 0–9 and days 9–17). **d** The total number of CFUs was counted by plating 1 × 10^4^ cells in methylcellulose. **e** Distribution of CFU subtypes. The bars indicate the mean ± SD; *p < 0.05, **p < 0.01 (control vs. CM 0–9 or CM 9–17). CFU-GM, granulocyte-macrophage.

PVCs create a specialized microenvironment that regulates the self-renewal and differentiation of HSCs in the BM^23^. A recent study reported that nonhematopoietic adipose tissue-derived PVCs support the long-term maintenance of human HSCs in vitro and improve their engraftment efficiency in vivo^24^. However, perivascular influences on hPSC-derived hematopoiesis have not been investigated. Thus, we investigated the effects of PVCs on the hematopoietic differentiation of hPSCs based on our optimized induction protocol. We isolated PVCs from HUCs (Supplementary Fig. 2) and attempted to treat hPSCs with PVC-CM during hematopoietic differentiation (days 0–17, CM 0–17). Unexpectedly, the percentages of hematopoietic progenitors and mature blood cells were significantly reduced in the PVC-CM-treated group compared to the control group (Supplementary Fig. 3). This result prompted us to treat hPSCs with PVC-CM at specific phases to determine whether PVC-CM is effective at the hemogenic specification phase (phase I, CM 0–9) or at the hematopoietic commitment phase (phase II, CM 9–17) (Fig. 4a). Notably, treatment with PVC-CM, compared with control treatment, during phase I significantly decreased the production of hematopoietic cells, whereas enhanced hematopoiesis was observed in cells treated with PVC-CM during phase II (Fig. 4b, c). The numbers of CFU-G, CFU-M and total CFUs in the population of HPCs generated from only phase II-treated cells were also significantly increased (Fig. 4d). However, the distribution of CFU subtypes in phase I- and phase II-treated cells was similar (Fig. 4e). These results indicated that PVCs exerted a positive influence on the hematopoietic differentiation of cells from hPSCs, especially during the commitment phase, via paracrine mechanisms.

### Treatment of hPSCs with β inducible gene H3 increased the output of hematopoietic colony-forming cells

**Fig. 5:**
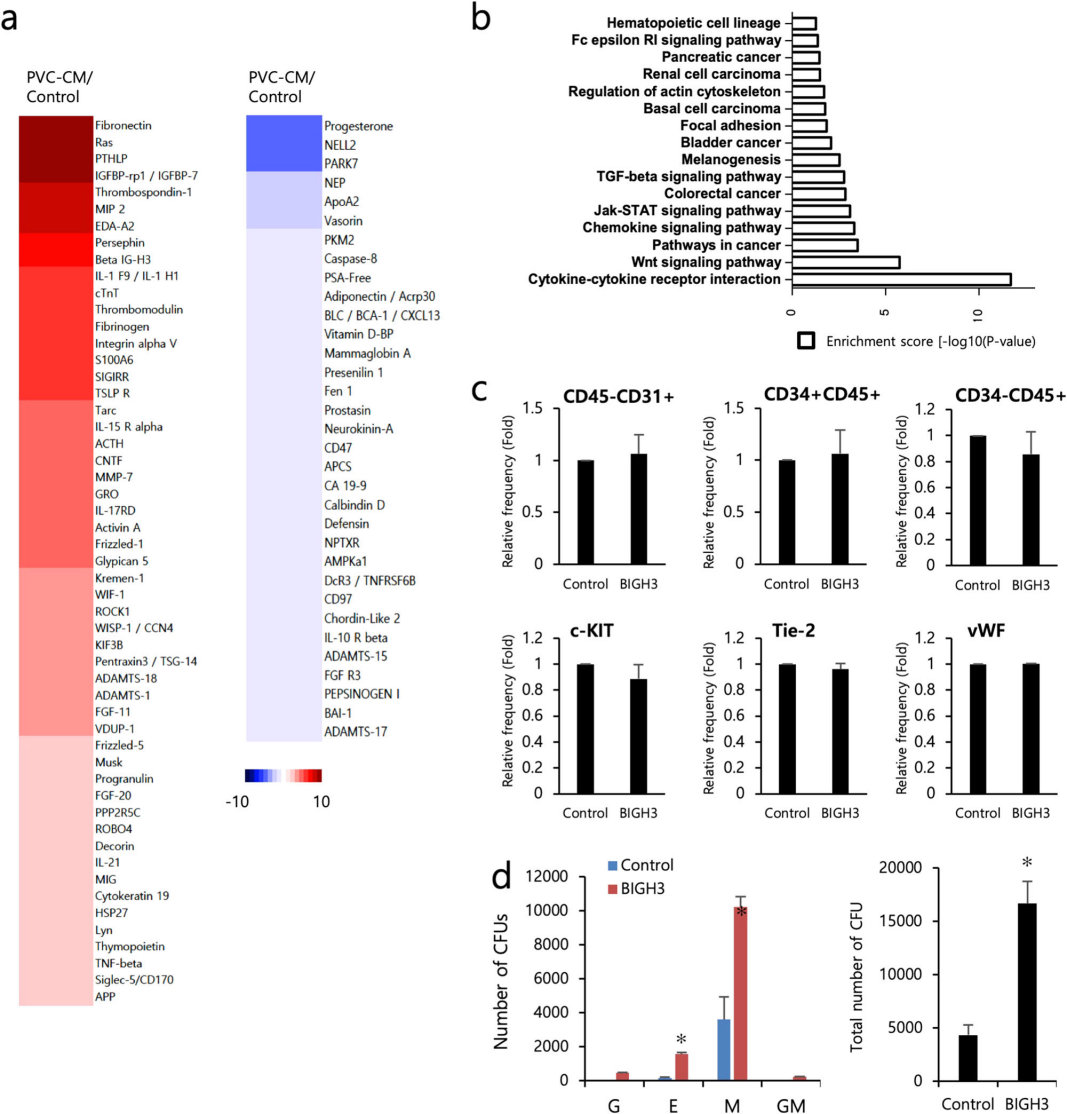
Protein array analysis reveals the secretion of ECM proteins, including BIGH3. **a** Heatmaps for upregulated and downregulated proteins in PVC-CM compared to the control. The color spectrum from blue to red indicates low to high expression. **b** Molecular and cellular functions associated with PVC-secreted proteins. **c** Flow cytometry (upper) and qRT-PCR (lower) analysis revealed that supplementation with BIGH3 during the hematopoietic commitment phase (days 9–17) did not enhance the production of hematopoietic or endothelial lineage cells. **d** Cultures differentiated with BIGH3 produced more CFU-E and CFU-M colonies than the control cultures. The bars indicate the mean ± SD; *p < 0.05.

To identify the secretion factors responsible for PVC-CM-enhanced hematopoietic differentiation, PVC-CM and serum-free non-CM as a control were subjected to protein array analyses. Secreted factors that showed ≥1.5-fold increase in expression in PVC-CM-treated cells relative to non-CM-treated control cells were considered to be significantly abundant. A protein array revealed 86 differentially expressed secretion factors (53 upregulated and 33 downregulated in PVC-CM) that are known to be involved in regulating the functions of cytokines and chemokines, specific signaling pathways, and hematopoietic cell lineage differentiation, between the two groups (Fig. 5a, b). Among the upregulated proteins in the PVC-CM group, the transforming growth factor-β inducible gene H3 (BIGH3), as part of the extracellular matrix (ECM) in the BM, contributes to the regulation of BM homeostasis by modulating the migration and adhesion of HSPCs; thus, we wanted to investigate its paracrine effect^25^. We treated cells with soluble BIGH3 (3 μg/mL) during the hematopoietic commitment phase and measured the frequencies of hematoendothelial lineage markers. We found that the addition of BIGH3 had no statistically significant effect on hematopoietic (CD34+CD45+, CD34−CD45+ and c-KIT) or endothelial (CD45−CD31+, Tie-2 and vWF) cell differentiation (Fig. 5c). However, BIGH3 augmented the total number of hematopoietic colony-forming cells by significantly increasing the numbers of CFU-E and CFU-M (Fig. 5d).

## Discussion

The robust and reproducible generation of hematopoietic lineage cells from hPSCs is dependent on the accurate in vitro recapitulation of the essential regulatory pathways that modulate HSC development in the early embryo and the adult BM^9,26^. Here, our study showed progress toward this goal, as we demonstrated the distinct temporal roles of BMP4 and the perivascular niche during hPSC-derived hematopoiesis.

BMP4 is one of the key hematopoiesis-inducing factors that has been widely used to differentiate hPSCs into cells of mesodermal and hematopoietic lineages^7,21^. Initial hematopoietic programs continuously applied BMP4 at low concentrations (20–50 ng/mL) from the mesoderm initiation phase to the hematopoietic maturation phase without considering the developmental stages^7,8,19^. However, a recent study reported that BMP4 signaling differentially affects hematopoietic output depending on the developmental stage^21^. HSCs that initially emerge in vivo in the AGM region are activated by BMP4, whereas BMP4-independent HSCs are predominant in the adult BM as developmental stages progress^27^. These findings indicate that there are variable BMP4 concentrations depending on HSC niches and the extent of response to BMP4 in HSC development changes^28^. Thus, we speculated that the duration and concentration of BMP4 exposure during hPSC-derived hematopoiesis has a decisive effect on hematopoietic output. In our study, a high initial dose of BMP4 treatment for 2 days robustly drove undifferentiated hPSCs to differentiate into cells of early primitive mesoderm and hematopoietic lineages, leading to lower hematopoietic variations among cell lines maintained under the E8+Vit condition compared with those maintained under the mTeSR+Mat condition. This advanced hematopoietic program might contribute to reduced variations in hematopoietic output from hPSCs due to donors from different genetic backgrounds, diverse cell types for iPSC generation, and reprogramming methods.

The perivascular niche, which partly includes mesenchymal stromal and endothelial cells in the BM, provides an important regulatory milieu in which HSPCs are maintained and undergo differentiation^23,24,29,30^. Recently, Corselli et al.^24^ reported that nonhematopoietic adipose tissue-derived PVCs support the long-term maintenance of human HSCs in vitro and improve their engraftment efficiency in vivo. HSC function and frequency in the BM are impaired by the deletion of the C-X-C motif ligand 12 and scf in PVCs^31,32^. We also found that alterations in BM hematopoietic composition due to hyperglycemic conditions can be restored by PVCs^17^. More recently, Comazzetto et al.^33^ demonstrated that scf from leptin receptor-expressing PVCs is a key factor in maintaining hematopoietic progenitors. Thus, we speculate that PVCs could affect the efficient induction of hPSCs to form cells of the hematopoietic lineage as well as the multilineage differentiation potential of hPSC-derived hematopoietic progenitors by mimicking a perivascular niche. Interestingly, we found that PVCs augmented the production of hematopoietic progenitors via paracrine mechanisms only when they were present during the hematopoietic commitment phase. Furthermore, perivascular niche-induced hematopoietic progenitors gave rise to more CFU-G and CFU-M colonies. Similarly, cocultures with endothelial niche cells significantly enhance the yield of hematopoietic progenitors and their hematopoietic activity, which is achieved by the activation of Notch signaling in hemogenic precursors^34,35^. However, Notch ligands, including JAG1 and DLL4, were not detected in our secretome analyses, suggesting that hematopoietic induction by the perivascular niche may not be due to Notch signaling. Instead, we found that several ECM proteins, including BIGH3, were significantly upregulated in PVC-CM compared with the control. BIGH3, as a part of the ECM in the BM, is known to control BM homeostasis by modulating the adhesion and migration of HSPCs^25^. However, the role of BIGH3 during hPSC-derived hematopoiesis is not known. Although BIGH3-stimulated hematopoietic progenitors gave rise to more CFU colonies, BIGH3 had no effect on their efficiency of differentiation into cells of hematopoietic and endothelial lineages. This may indicate that BIGH3 alone is insufficient to stimulate downstream signals to enhance the hematopoietic commitment of hPSCs. BIGH3 interacts with other ECM proteins, including integrins, fibronectin and collagens, and can also function as a linker protein between the ECM and integrins under various physiological and pathological conditions^36,37^. These findings suggest that cotreatment with BIGH3 and other ECM proteins that have been confirmed to aid hematopoietic commitment might be useful in the activation and internalization of BIGH3 for the control hematopoietic lineage-specific genes.

Our study underscores the importance of spatiotemporal regulation for the efficient and reproducible derivation of hematopoietic cells from hPSCs in vitro. We believe that our optimized system, which involves serum-, feeder- and xeno-free conditions throughout the procedure of differentiation with perivascular niche factors, will be a robust strategy to generate more functional and clinically relevant HSPCs with therapeutic potential.

## Supplementary information

Supplementary Information
