# Supplementary material for: BMP4 and perivascular cells promote hematopoietic differentiation of human pluripotent stem cells in a differentiation stage-specific manner
Source: Exp Mol Med. 2020 Jan 20;52(1):56–65. doi: 10.1038/s12276-019-0357-5 (PMC7000736; doi:10.1038/s12276-019-0357-5)
Supplement: Supplementary file 1 — Supplementary Information [file 12276_2019_357_MOESM1_ESM.docx]

**Supplementary Information**

**BMP4 and perivascular cells promote hematopoietic differentiation of human pluripotent stem cells in a differentiation stage-specific manner**

Suji Jeong^1^, Borim An^1^, Jung-Hyun Kim^1^, Hyo-Won Han^2^, Jung-Hyun Kim^2^, Hye-Ryeon Heo^1^, Kwon-Soo Ha^3^, Eun-Taek Han^4^, Won Sun Park^5^, Seok-Ho Hong^1^*

^1^*Department of Internal Medicine, School of Medicine, Kangwon National University, Chuncheon, 24341 Republic of Korea*

*^2^Dvision of Intractable Diseases, Center for Biomedical Sciences, Korea National Institute of Health, Korea Centers for Disease Control and Prevention, Cheongju 28159, Republic of Korea*

^3^*Department of Molecular and Cellular Biochemistry, School of Medicine, Kangwon National University, Chuncheon, 24341Republic of Korea*

^4^*Department of Medical Environmental Biology and Tropical Medicine, School of Medicine, Kangwon National University, Chuncheon, 24341 Republic of Korea*

^5^*Department of Physiology, School of Medicine, Kangwon National University, Chuncheon, 24341 Republic of Korea*

*Kangwon National University, Chuncheon, 24341 Republic of Korea*


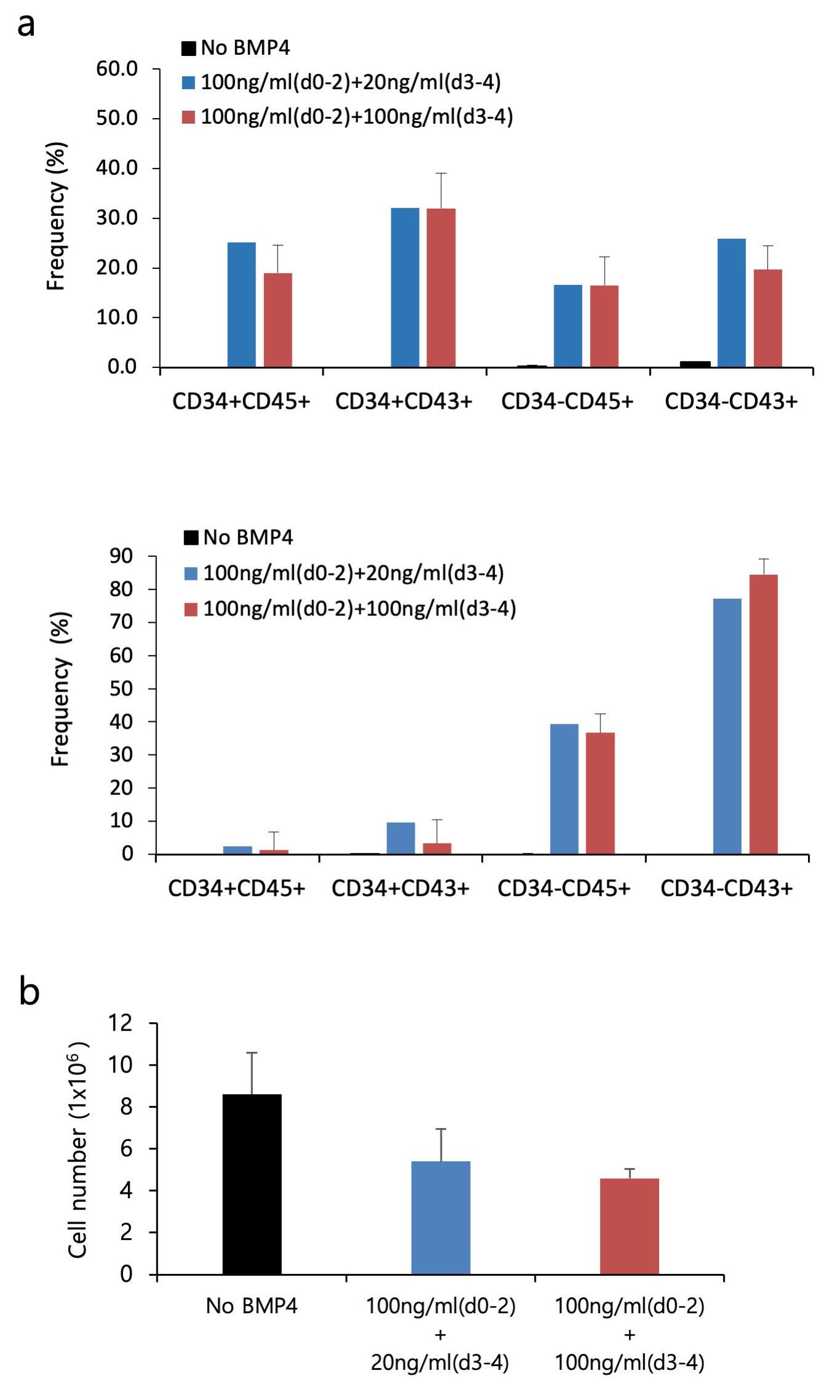


**Supplementary** **Fig. 1 Effects of the duration of BMP4 treatment on hematopoietic differentiation. a** Comparison of the length (day 0-2 vs day 0-4) of BMP4 (100 ng/mL) exposure to hPSCs during early mesodermal induction for hematopoietic output. **b** Total cell number was counted on day 16 of hematopoietic differentiation. Bars indicate mean±SD.

**
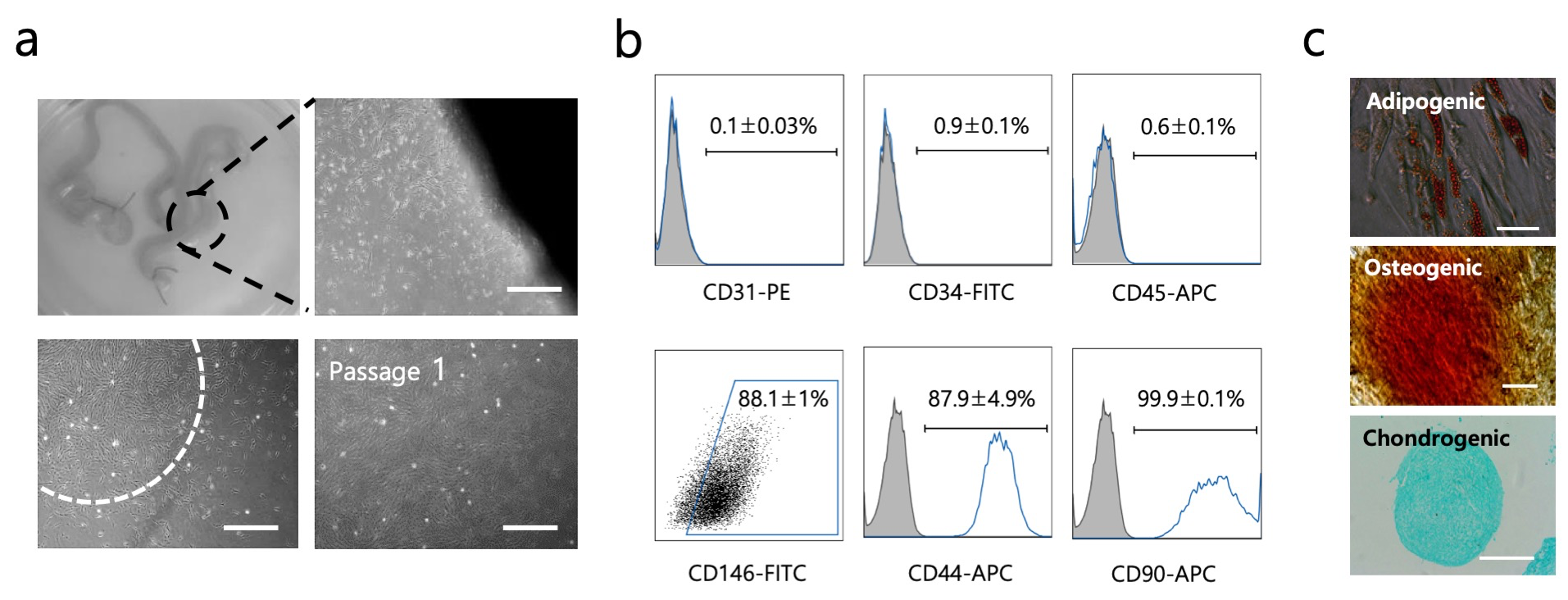
**

**Supplementary** **Fig. 2 Isolation and characterization of PVCs derived from vessels of HUCs. a** Isolation of PVCs derived from vessels of HUCs. Scale bar, 100 μm. **b** Phenotypic characterization of the isolated PVCs was measured by flow cytometry. Frequencies in histograms indicate mean±SD. **c** Functional PVCs able to differentiate into adipogenic, osteogenic and chondrogenic cells. Scale bar, 100 μm, 200 μm.

**
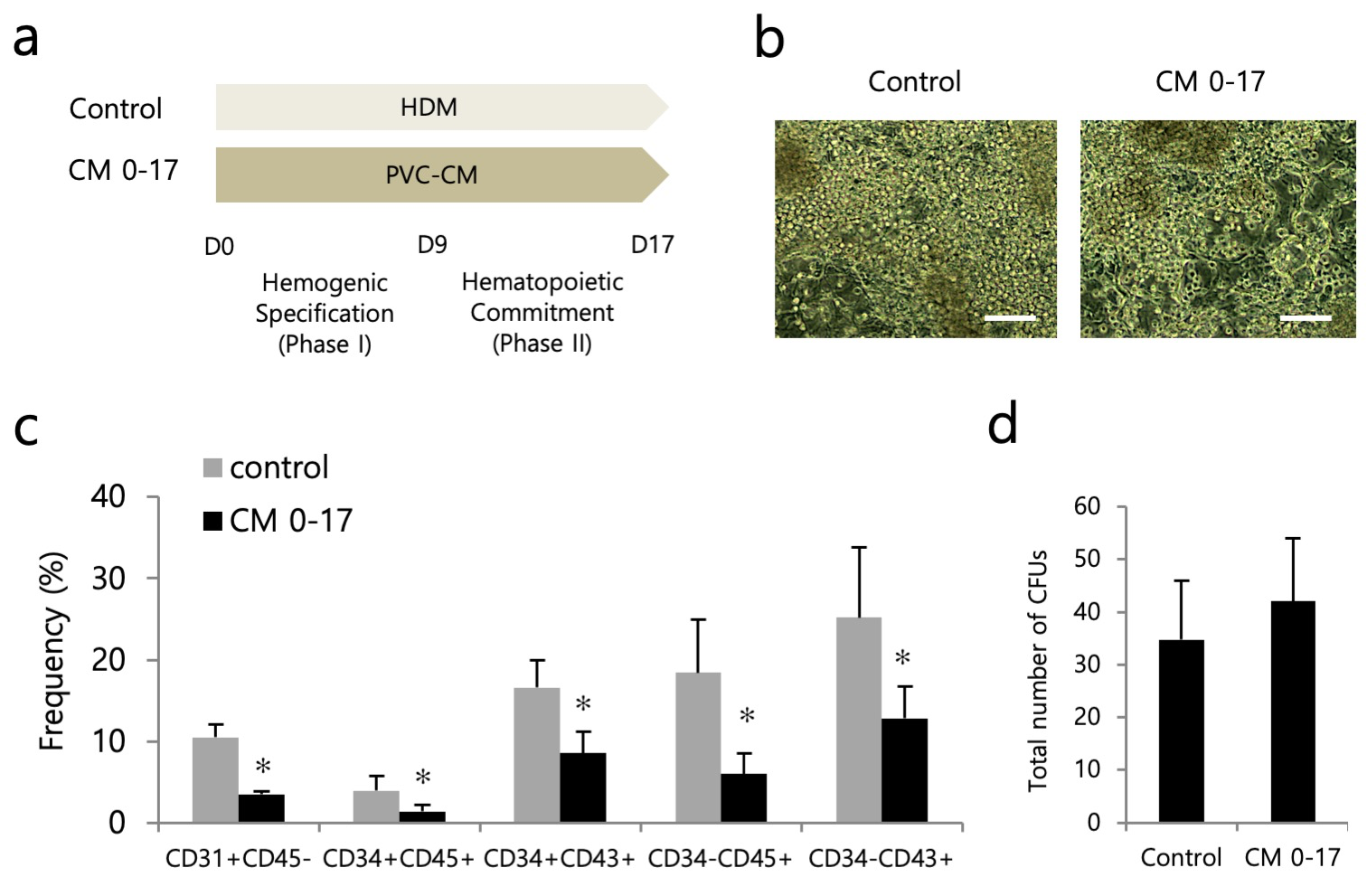
**

**Supplementary** **Fig. 3 Effect of PVC on hPSC-derived hematopoiesis. a** Experimental scheme to determine the paracrine effects of PVCs on hematopoietic differentiation of hPSCs (CHA15 and iPS-NT4-S1). **b** Representative bright field images of colonies at day 17 of hematopoietic differentiation. Scale bar, 100 μm. **c** Effects of PVC-CM on the production of hematopoietic lineage cells. **d** The total number of CFUs was counted by plating 1×10^4^ cells into methylcellulose. Bars indicate mean±SD. **p*<0.05 (Control vs. CM 0-17).
